# The Optimal Surgical Strategy and Dose for Esotropia

**DOI:** 10.7759/cureus.115667

**Published:** 2026-09-02

**Authors:** Christopher T Leffler, Emilia Varrone, Satya S Paruchuri, Catherine Phan

**Affiliations:** 1 Ophthalmology, Virginia Commonwealth University, Richmond, USA

**Keywords:** esotropia, optimum, strabismus, strabismus surgery, surgical dose

## Abstract

The study objective was to determine the clinical predictors of surgical failures following horizontal strabismus surgery for esotropia, in order to estimate the model-predicted optimal surgical strategy. A retrospective pooled observational case series of published cases was performed. Patients having horizontal strabismus surgery for esotropia, published between 1940 and 2025, with known preoperative deviation, surgical approach, and outcome were studied. Clinical data from individual patients having strabismus surgery for esotropia were recorded from published case series and analyzed using multivariable logistic regression to predict over- and under-correction. The main outcome measure was surgical failure, as determined by reoperation, suture adjustment, or postoperative strabismus of 10 prism diopters (PD) or more. We abstracted individual patient data for 3518 surgeries from 163 publications. Binocular (as compared with monocular) surgery was associated with fewer under-corrections (odds ratio (OR): 0.75, 95% confidence interval (CI): 0.61 to 0.92, p=0.005) and more over-corrections (OR: 1.87, 95% CI: 1.26 to 2.79, p=0.002, n=3266). Increasing preoperative deviation was associated with more under-corrections (OR: 1.06/°, 95% CI: 1.05/° to 1.07/°, p<0.0001) and fewer over-corrections (OR: 0.97/°, 95% CI: 0.95/° to 0.99/°, p=0.001, n=3266). Increasing surgical dose was associated with fewer under-corrections (OR: 0.95/mm, 95% CI: 0.91/mm to 0.99/mm, p=0.01) and more over-corrections (OR: 1.08/mm, 95% CI: 1.01/mm to 1.16/mm, p=0.02, n=3266). The failure rate was minimized with a large per-muscle surgical dose. As the preoperative deviation increases, one progresses from unilateral recessions, to unilateral recession-resections, and then to bi-medial recessions. Under a range of assumptions, bi-medial recessions of 6 mm are optimal (according to the models) for preoperative deviations of 45-50 prism diopters. We conclude that larger doses for esotropia surgery do produce a larger response. Most models predicted the lowest failure rates with large recessions or resections, with additional muscles operated for larger preoperative deviations.

## Introduction

For decades, strabismus surgeons have debated the preferred surgical strategy and dose for esotropia. Some, such as Richard Scobee in 1951, have recommended reasonably large surgeries in all cases (e.g., 6 mm medial rectus recessions), with the only variation being the number of muscles operated [1]. Others, such as Marshall Parks in 1975, recommended two-muscle surgeries in all cases, but with graded surgical dosing, with smaller doses used for smaller angles of deviation [2].

One conventional approach to predict the likely outcome of strabismus surgery has been to look at the change in alignment as a function of the surgical dose measured in mm. However, it has been pointed out that the major predictor of the change in alignment is the preoperative alignment itself [3]. Moreover, it is conventional for surgeons to deliver a larger surgical dose when the preoperative deviation is greater [2]. Therefore, both change in alignment and surgical dose will be correlated with preoperative alignment, and, hence, with each other, even if the surgical dose does not change the size of the response in a causative fashion. In other words, spurious dose-response relations can be generated, even with small datasets [3].

Recent approaches to understand strabismus surgery failures have looked at registries or insurance datasets with thousands of patients [4-9]. Unfortunately, these large datasets have not included preoperative or postoperative deviations, or the surgical dose in mm [4-9]. This fact has limited their ability to offer insight into the preferred surgical approach.

Therefore, we abstracted and analyzed individual patient data from case series published from 1940 to 2025 to determine predictors of over- and under-correction following surgery for esotropia and to better understand the preferred surgical strategy and dose.

## Materials and methods

Studies of patients having medial rectus recessions and/or lateral rectus resections for esotropia were identified in Google Scholar by searching for terms such as “esotropia”, “medial rectus recession”, and “lateral rectus resection”, combined with “mm”, in October 2025, as tabulated in Table 1.

**Table 1:** Search strategy to identify candidate studies in October 2025

intitle:unilateral intitle:"medial rectus" recession intitle:esotropia	33
intitle:"medial rectus" recession intitle:esotropia	202
intitle:"medial rectus" intitle:recession intitle:esotropia	148
esotropia "medial rectus recession" "lateral rectus resection" table	633
esotropia "medial rectus recession" -"lateral rectus resection" table	1380
esotropia mm "lateral rectus resection" table -"medial rectus recession"	87
esotropia mm "bimedial recession" -"lateral rectus resection" table -"medial rectus recession"	95

We identified qualifying studies from 771 candidate studies for review from 1940 to 2025. The studies included and excluded, primarily for lacking individual patient information with adequate specificity, such as the mm of surgery, are listed at https://doi.org/10.13140/RG.2.2.30190.37445. We screened 771 reports. Study inclusion criteria included the following: individual patient data (preoperative deviation, mm of surgery performed, postoperative status as success, and over- or under-correction), relevant information provided in the English language, and at least two patients with strabismus. We excluded 608 studies for the following reasons: studies of botulinum toxin injection (n=7), individual case reports (n=16), reviews (n=40), studies of exotropia (n=16), studies lacking individual patient data (n=457), nonsurgical treatment (n=4), relevant information not in the English language (n=1), cases selected on the basis that they succeeded or failed, nonrepresentative (n=11), use of posterior fixation sutures (n=8), pulley surgery (n=4), surgery on a previously operated muscle (n=8), retracted study (n=1), slanting recessions (n=6), transposition surgery (n=16), union surgeries (n=3), vertical surgery (n=6), and Y-splitting surgery (n=4). Each excluded report was assigned a single primary reason for exclusion. Steven Archer provided us with raw data on strabismus outcomes without any individually identifiable patient information [10].

The literature search was designed to identify published reports containing extractable individual patient-level surgical and outcome data and was not intended as a formal systematic review of the entire esotropia surgical literature.

Study setting, duration, and timeline

This was a retrospective pooled analysis of individual patient data abstracted from case series previously published from 1940 through 2025. The literature search was performed in October 2025, and data were analyzed in October and November 2025.

Under-corrections and over-corrections were defined as deviations of 10 prism diopters (PD) or more of esotropia or exotropia postoperatively, a reoperation, or need for adjustment for esotropia or exotropia. For patients with a preoperative deviation of 10 prism diopters or less, failure was additionally defined as failure to improve the deviation postoperatively. For example, we classified a patient with 6 prism diopters of preoperative esotropia who had a postoperative deviation of 8 prism diopters of esotropia or exotropia as a surgical failure because the patient experienced no improvement in ocular alignment. This criterion prevented an unchanged or worsened deviation of less than 10 prism diopters from being classified as a surgical success solely because it remained below the conventional 10-prism-diopter failure threshold. The follow-up date in the study is the same as the outcome date used for the data analysis. For studies that reported recessions of the medial rectus muscle as distance from the limbus, the original insertion distance was assumed to be 5.5 mm. As with previous studies, we abstracted data from figures when not tabulated [3].

Multivariable logistic regression models to predict under- and over-corrections were prepared with the following variables: (1) preoperative deviation in degrees (°) (when both far and near deviations were provided, the larger deviation was used, and when deviations with and without refractive correction were provided for accommodative esotropia, the deviation with refractive correction was used), (2) the total number of horizontal rectus muscles operated and (3) binocular versus monocular surgery. Variables tested in the multivariable models included those significant in univariate analysis, as well as the total surgical dose (mm). The surgical dose was defined as the sum of recessions and resections in mm.

We explored the effect on the model-predicted optimal surgical strategy if over-corrections are more undesirable than under-corrections, perhaps because under-corrections are less likely than over-corrections to produce more severe adverse outcomes, such as a failure of ductions. We also determined for maximal surgery the preoperative deviation for which at least 20 and at least 40 cases had been performed. Statistical analysis was performed with XLSTAT version 2025.1. The study was approved by the Virginia Commonwealth University Office of Research Subjects Protection (approval number: HM300000233).

## Results

Patient and surgery characteristics

We identified 163 qualifying reports containing 3518 surgeries in 3504 patients.

Most analyses focused on 3266 cases without thyroid disease, for which, if adjustable sutures were used, the initial alignment (before adjustment) was known (Tables 2-4). Only 62 of the surgeries in these baseline analyses used adjustable sutures.

**Table 2:** Univariate predictors of under-corrections from strabismus surgery for esotropia Totals vary by row due to missing or ambiguous data N=3266 cases, including 62 adjustable cases and no thyroid eye disease cases PD: prism diopters

Factor	Factor present			Factor absent			p value
	Under-corrected (%)	Under-corrected (number)	Total	Under-corrected (%)	Under-corrected (number)	Total	
Child (age < 18 years)	26.7%	673	2516	35.9%	132	368	<0.001
Child < 6 years old	22.5%	356	1579	30.2%	294	975	<0.001
Age ≥ 45 years	21.7%	25	115	27.4%	803	2931	0.20
Binocular surgery	26.7%	561	2098	28.5%	333	1168	0.29
1-muscle surgery	21.3%	138	649	28.9%	756	2617	<0.001
3- or 4-muscle surgery	18.5%	50	271	28.2%	844	2995	<0.001
4-muscle surgery	30.2%	16	53	27.3%	878	3213	0.64
Total surgery ≥ 11 mm	29.3%	491	1678	25.4%	403	1588	0.06
Preoperative deviation ≥ 38 PD	33.1%	537	1623	21.7%	357	1643	<0.001
Follow-up ≥ 6 months	26.3%	542	2063	31.3%	324	1036	0.004
Duane syndrome	18.5%	23	124	27.8%	853	3064	0.02

**Table 3:** Univariate predictors of over-corrections from strabismus surgery for esotropia Totals vary by row due to missing or ambiguous data N=3266 cases, including 62 adjustable cases and no thyroid eye disease cases PD: prism diopters

Factor	Factor present			Factor absent			p value
	Over-corrected (%)	Over-corrected (number)	Total	Over-corrected (%)	Over-corrected (number)	Total	
Child (age < 18 years)	7.4%	186	2516	4.6%	17	368	0.06
Child < 6 years old	8.0%	126	1579	6.5%	63	975	0.16
Age ≥ 45 years	8.7%	10	115	6.7%	196	2931	0.35
Binocular surgery	8.5%	179	2098	3.3%	39	1168	<0.001
1-muscle surgery	2.5%	16	649	7.7%	202	2617	<0.001
3- or 4-muscle surgery	15.1%	41	271	5.9%	177	2995	<0.001
4-muscle surgery	24.5%	13	53	6.4%	205	3213	<0.001
Total surgery ≥ 11 mm	8.0%	134	1678	5.3%	84	1588	0.002
Preoperative deviation ≥ 38 PD	7.7%	125	1623	5.7%	93	1643	0.02
Follow-up ≥ 6 months or more	7.0%	145	2063	5.5%	57	3099	0.11
Duane syndrome	2.4%	3	124	6.9%	210	3064	0.06

**Table 4:** Multivariable logistic predictors of under-corrections and over-corrections after strabismus surgery for esotropia Probability of under-correction = 1 / (1 + exp(-(-1.447552+0.055703*(preoperative deviation in degrees) -0.053285*(total surgery in mm) -0.291698*(binocular surgery)-0.024721*(number of muscles operated)))) Probability of over-correction = 1 / (1 + exp(-(-4.195557-0.033682*(preoperative deviation in degrees) +0.079761*(total surgery in mm) +0.626323*(binocular surgery)+0.474277*(number of muscles operated)))) The variable binocular surgery equals 1 for binocular surgery and 0 for monocular surgery. N=3266 cases, including 62 adjustable cases and no thyroid eye disease cases

Variable	Under-corrections				Over-corrections			
	Odds ratio			p value	Odds ratio			p value
	Estimate	Lower 95%	Upper 95%		Estimate	Lower 95%	Upper 95%	
Preoperative deviation (°)	1.06	1.05	1.07	<0.0001	0.97	0.95	0.99	0.001
Surgical dose (mm)	0.95	0.91	0.99	0.01	1.08	1.01	1.16	0.02
Number of muscles operated	0.98	0.75	1.26	0.85	1.61	1.07	2.41	0.02
Binocular surgery	0.75	0.61	0.92	0.005	1.87	1.26	2.79	0.002

The mean preoperative deviation was 22.3° (standard deviation (SD): 9.7°) (which corresponds with 40.9 PD), with a range of 0° to 52° (0 to 128 PD, n=3266). (Some strabismus surgeries are performed on patients with orthophoria in primary gaze but esotropia in lateral gaze due to incomitance, or for accommodative esotropia seen only without correction [11-13]).

The surgical approach was bi-medial rectus (n=1616 surgeries), unilateral medial rectus (n=572), both horizontal muscles in one eye (n=517), bilateral lateral rectus (n=224), bi-medial rectus with one lateral rectus (n=205), unilateral lateral rectus (n=79), all four horizontal muscles (n=50), or bilateral lateral rectus muscle with one medial (n=3). Of 2963 surgeries that involved at least one medial rectus recession, 2361 (80%) were between 3 mm and 6 mm, inclusive.

Associations with over- and under-correction

Binocular (as compared with monocular) surgery was associated with fewer under-corrections (odds ratio (OR): 0.75, 95% confidence interval (CI): 0.61 to 0.92, p=0.005) and more over-corrections (OR: 1.87, 95% CI: 1.26 to 2.79, p=0.002, n=3266, Table 4). Increasing preoperative deviation was associated with more under-corrections (OR: 1.06/°, 95% CI: 1.05/° to 1.07/°, p<0.0001) and fewer over-corrections (OR: 0.97/°, 95% CI: 0.95/° to 0.99/°, p=0.001). Increasing surgical dose was associated with fewer under-corrections (OR: 0.95/mm, 95% CI: 0.91/mm to 0.99/mm, p=0.01) and more over-corrections (OR: 1.08/mm, 95% CI: 1.01/mm to 1.16/mm, p=0.02). For a given surgical dose in mm, operating on more muscles increased the likelihood of over-corrections (OR: 1.61/muscle, 95% CI: 1.07/muscle to 2.41/muscle, p=0.02, Table 4). This model included no cases of thyroid eye disease (n=3266, Table 4).

Most surgeries (87.2%, 2516 of 2884 surgeries with data available) were performed in children under age 18 years. In univariate analysis, children were less likely to be under-corrected (p<0.001, Table 2) and tended to have more over-corrections, although not significantly so (p=0.06, Table 3). In multivariable logistic regression, children had a lower probability of under-correction (OR: 0.59, 95% CI: 0.46 to 0.76, p<0.001, n=2884).

Postoperative follow-up was 6 months or more in 2063 of 3099 surgeries with data available (66.6%). In univariate analysis, follow-up of 6 months or more was associated with fewer under-corrections (26.3% versus 31.3%, p=0.004, Table 2), but only a nonsignificant trend toward more over-corrections (7.0% versus 5.5%, p=0.11, Table 3). With multivariable logistic regression (n=3099), follow-up over 6 months was associated with fewer under-corrections (OR: 0.78, 95% CI: 0.66 to 0.92, p=0.004). These observations are consistent with an exotropic drift after surgery for esotropia.

Duane syndrome was present in 124 (3.8%) surgeries. In univariate analysis, Duane syndrome was associated with a lower risk of under-correction (18.5% versus 27.8%, p=0.02, Table 2). However, in a multivariable logistic regression model, the association of Duane syndrome with under-corrections was no longer significant (OR: 0.69, 95% CI: 0.43 to 1.11, p=0.13, n=3188).

Preferred surgical approach

From the equations for the probability of under- and over-corrections, the total failure rate can be estimated. The model-predicted optimal surgical strategy is that which minimizes the failure rate. For instance, for bi-medial recessions, if under- and over-corrections are considered equally undesirable, the lowest failure rate for 20 prism diopters of esotropia occurs with 5 mm recessions for both eyes (Figure 1).

**Figure 1:**
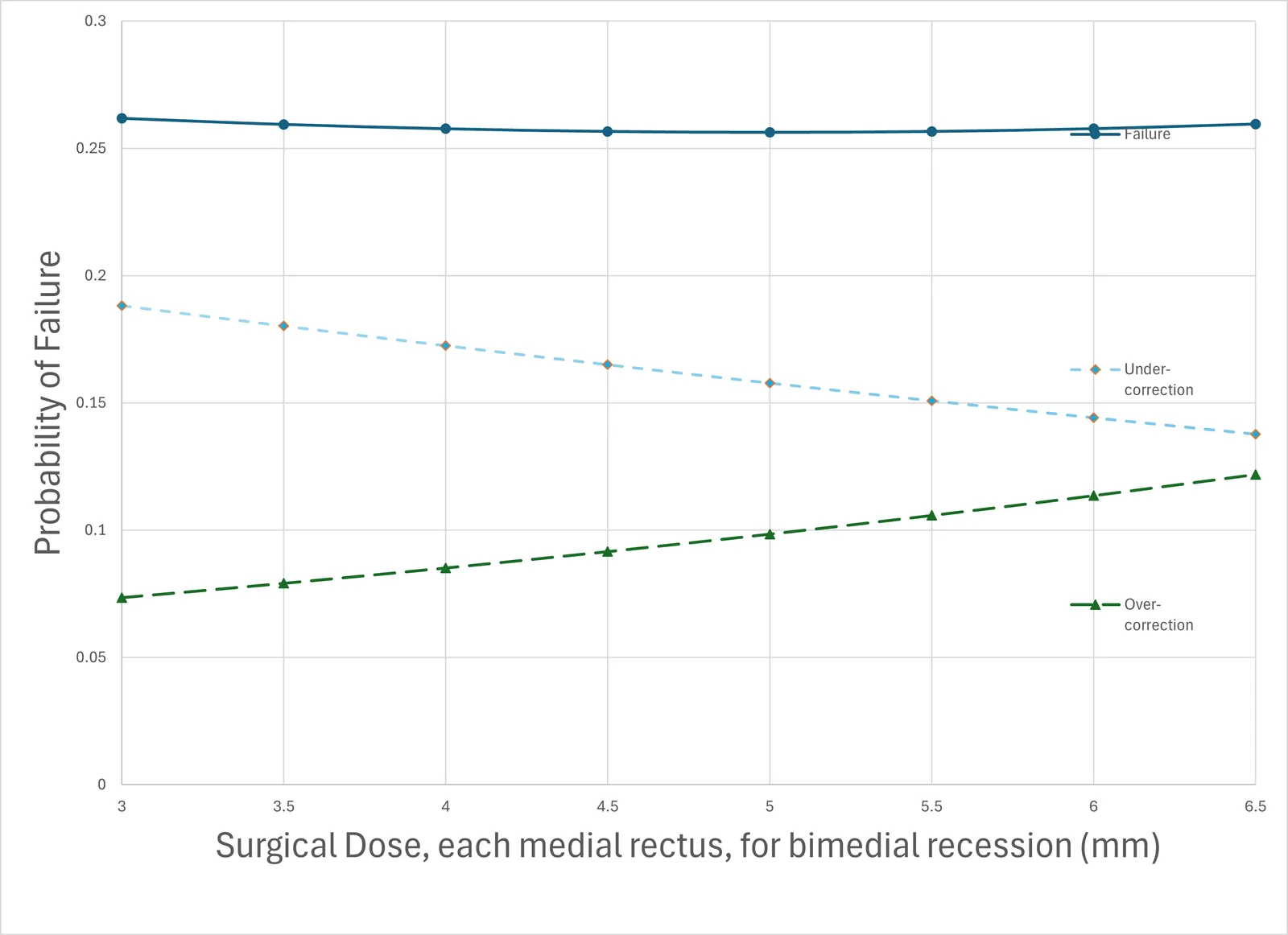
Probability of failures, under-corrections, and over-corrections for bi-medial recession with a preoperative deviation of 20 prism diopters, as a function of surgical dose in mm per muscle This is the basic model in which under- and over-corrections are considered equally undesirable.

For monocular surgeries, the models predicted maximal surgical doses for a deviation of 20 prism diopters (Figures 2, 3).

**Figure 2:**
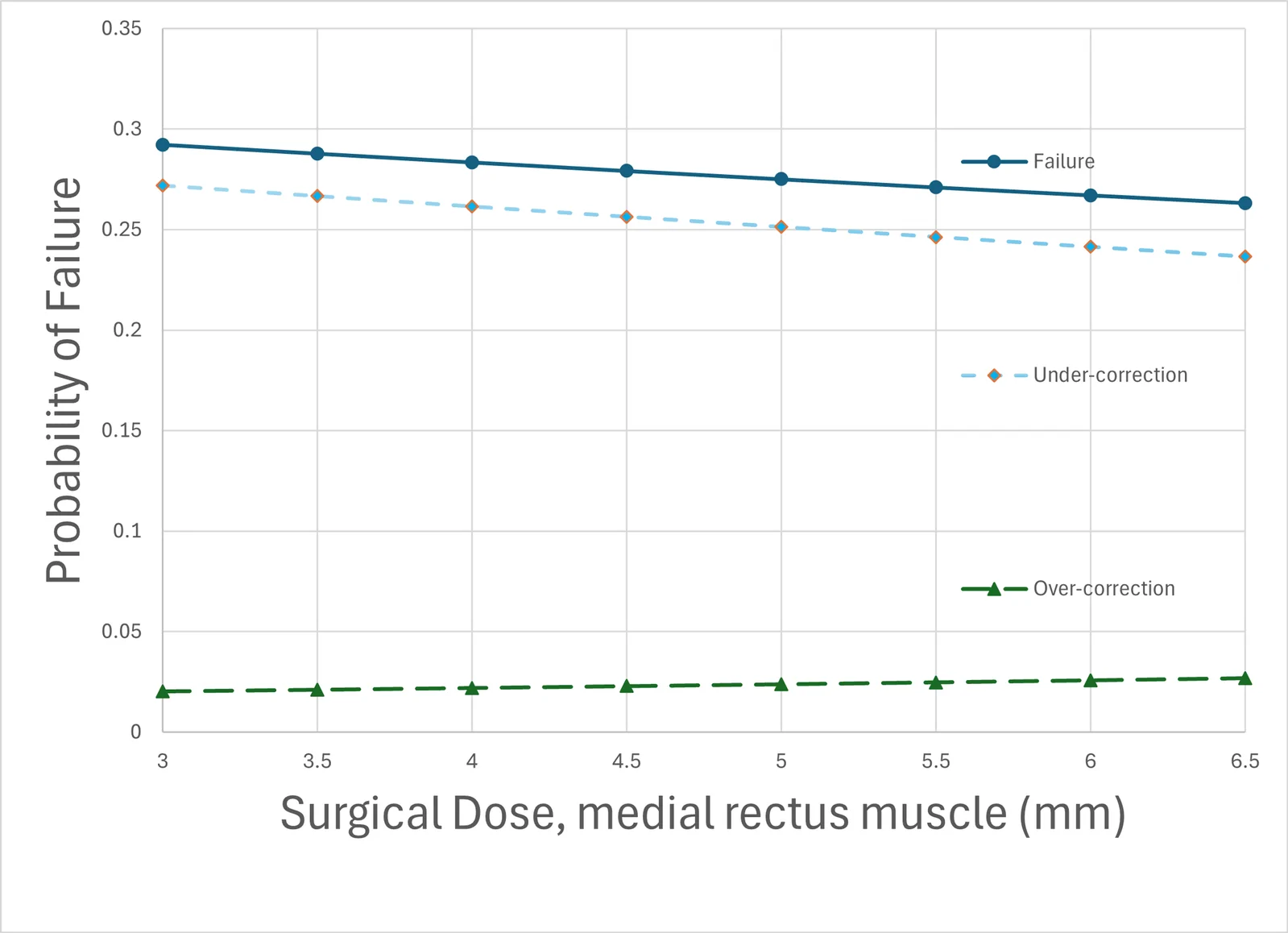
Probability of failure, under-correction, and over-correction for unilateral medial rectus recession for 20 PD preoperative deviations as a function of surgical dose in mm, for the basic model, in which under- and over-corrections are considered equally undesirable PD: prism diopters

**Figure 3:**
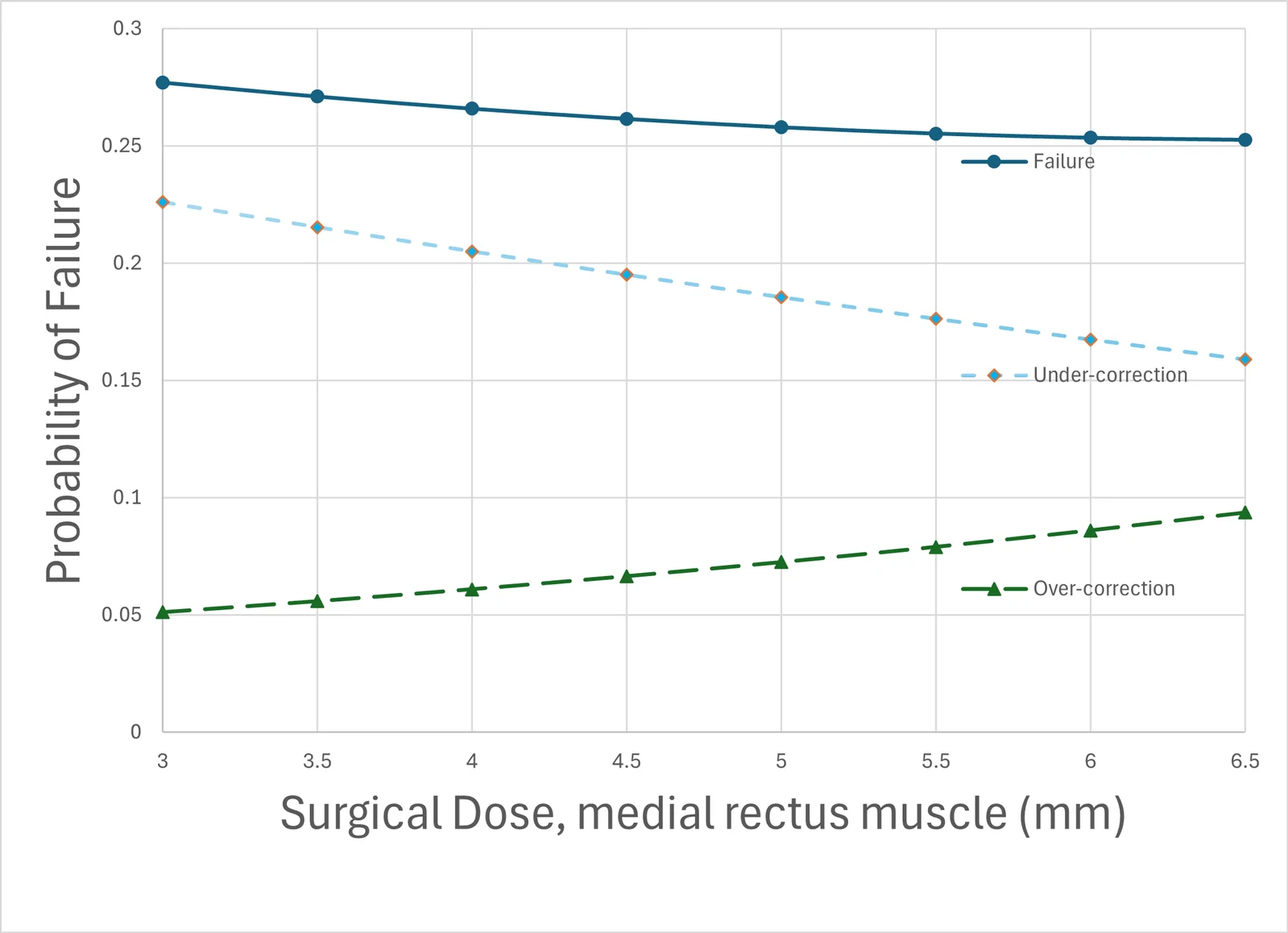
Probability of failure, under-correction, and over-correction for unilateral two-muscle strabismus surgery for 20 PD preoperative deviations as a function of surgical dose for medial rectus recession plus lateral rectus resection, for the model in which under- and over-corrections were considered equally undesirable The resections were one-third larger than the recessions. PD: prism diopters

Although the precise model-predicted optimal surgical approach depends on the specific assumptions, certain common themes can be identified. If one must perform two-muscle surgery, as in the tables of Parks (1975) [2], then larger preoperative deviations may require larger surgical doses, measured in mm (Tables 5, 6). The relative value that one places on over-corrections and under-corrections influences the surgical dose estimated to be optimal in the models. For instance, assuming that under-corrections only result in significant adverse findings 64.5% as often as over-corrections results in tables that approximate the bi-medial recession tables of Parks (1975) [2], with model-predicted optimal doses of 4 mm OU for 25 PD and 4.5 mm OU for 30 PD (Table 5).

**Table 5:** Bi-medial recession tables The “minimize failure rate” model weights under-corrections and over-corrections as equally undesirable. The subsequent models considered under-corrections to be 64.5% and 40% as likely as over-corrections to result in severe adverse outcomes. With a weight of 64.5%, the tables approximate the bi-medial recession tables of Parks (1975). Values from 3.0 to 6.0 mm were tested in the model. PD: prism diopters

Preoperative deviation (PD)	Medial rectus recession, both eyes, mm (each muscle)		
	Minimize failure rate	Under-corrections weighted 64.5%	Under-corrections weighted 40%
≤10	3.0	3.0	3.0
15	4.0	3.0	3.0
20	5.0	3.0	3.0
25	6.0	4.0	3.0
30	6.0	4.5	3.0
35	6.0	5.5	3.0
40	6.0	6.0	4.0
45	6.0	6.0	5.0
50	6.0	6.0	6.0

**Table 6:** Unilateral medial rectus recession plus lateral rectus resection The “minimize failure rate” model weights under-corrections and over-corrections as equally undesirable. The subsequent models considered under-corrections to be 64.5% and 40% as likely as over-corrections to result in severe adverse outcomes. With a weight of 40%, the table approximates the unilateral recess/resect table of Parks (1975). Medial rectus recessions from 3.0 to 6.0 mm were tested in the model, with the lateral rectus resections one-third larger than the recessions. All recession and resection values in millimeters (mm) MR: medial rectus, LR: lateral rectus

Preoperative deviation (prism diopters)	Minimize failure rate		Under-corrections weighted 64.5%		Under-corrections weighted 40%	
	MR recess	LR resect	MR recess	LR resect	MR recess	LR resect
0	4.0	5.3	3.0	4.0	3.0	4.0
5	5.0	6.7	3.0	4.0	3.0	4.0
10	5.5	7.3	4.0	5.3	3.0	4.0
15	6.0	8.0	4.5	6.0	3.0	4.0
20	6.0	8.0	5.5	7.3	3.5	4.7
25	6.0	8.0	6.0	8.0	4.0	5.3
30	6.0	8.0	6.0	8.0	5.0	6.7
35	6.0	8.0	6.0	8.0	6.0	8.0

Moreover, assuming that under-corrections only result in significant adverse findings 40% as often as over-corrections approximates the unilateral recess/resect tables of Parks (1975) [2], with a model-predicted optimal dose of 4 mm recession/6 mm resection for 25 PD (Table 6).

If the restriction that surgery must involve two muscles is removed, then all the models predict maximal (or near-maximal) surgery to minimize the failure rate. One does not perform small recessions or resections. Rather, if one decides to operate on a muscle, one performs a reasonably large surgery, in accord with the approach of Scobee (1951) [1]. If the deviation is small, one operates on few muscles, and if large, one operates on more muscles. Moreover, there is a consistent progression as one moves from smaller to larger preoperative deviations: (1) unilateral recession, (2) unilateral recess-resect, (3) bi-medial recession, (4) three-muscle surgery, and (5) four-muscle surgery (Table 7, Figure 4). Finally, certain preoperative deviations have the same approach regardless of the weighting assumptions used. For instance, preoperative deviations of 45 to 50 prism diopters are optimally treated (according to the models) with 6-mm bi-medial recessions in all models tested.

**Table 7:** Model-predicted optimal surgical approach for esotropia The basic model minimizes the raw failure rate and considers under- and over-corrections equally undesirable. The weighted model assumes under-corrections are only 64.5% or 40% as likely to produce severe adverse outcomes as over-corrections. These assumptions produce model-predicted optimal bi-medial recession and unilateral recess/resect tables, respectively, which approximate the Parks tables. The weighted plus threshold model also requires that operation on each additional muscle must decrease the failure rate by 2% to justify the added surgical risk. MR: medial rectus, LR: lateral rectus, "-": not applicable, PD: preoperative deviation in prism diopters

Number of muscles	Surgical approach	Basic model		Under-corrections weighted 64%		Under-corrections weighted 64% + 2% threshold		Under-corrections weighted 40%	
		Lower PD	Upper PD	Lower PD	Upper PD	Lower PD	Upper PD	Lower PD	Upper PD
1	MR recess 5 mm	-	-	-	-	-	-	0	0
	MR recess 6 mm	≥0	≤5	≥0	≤20	≥0	≤30	≥5	≤30
2	MR recess 5.5 mm + LR resect 7.3 mm	≥10	≤10	-	-	-	-	-	-
	MR recess 6 mm + LR resect 8 mm	≥15	≤30	≥25	≤40	≥35	-	≥35	≤40
	MR recess 6 mm OU	≥35	≤50	≥45	≤70	≥45	≤120	≥45	≤120
3	MR recess 6 mm OU + LR resect 8 mm	≥55	≤80	≥75	≤120	-	-	-	-
4	MR recess 6 mm OU + LR resect 8 mm OU	≥85	≤120	-	-	-	-	-	-

**Figure 4:**
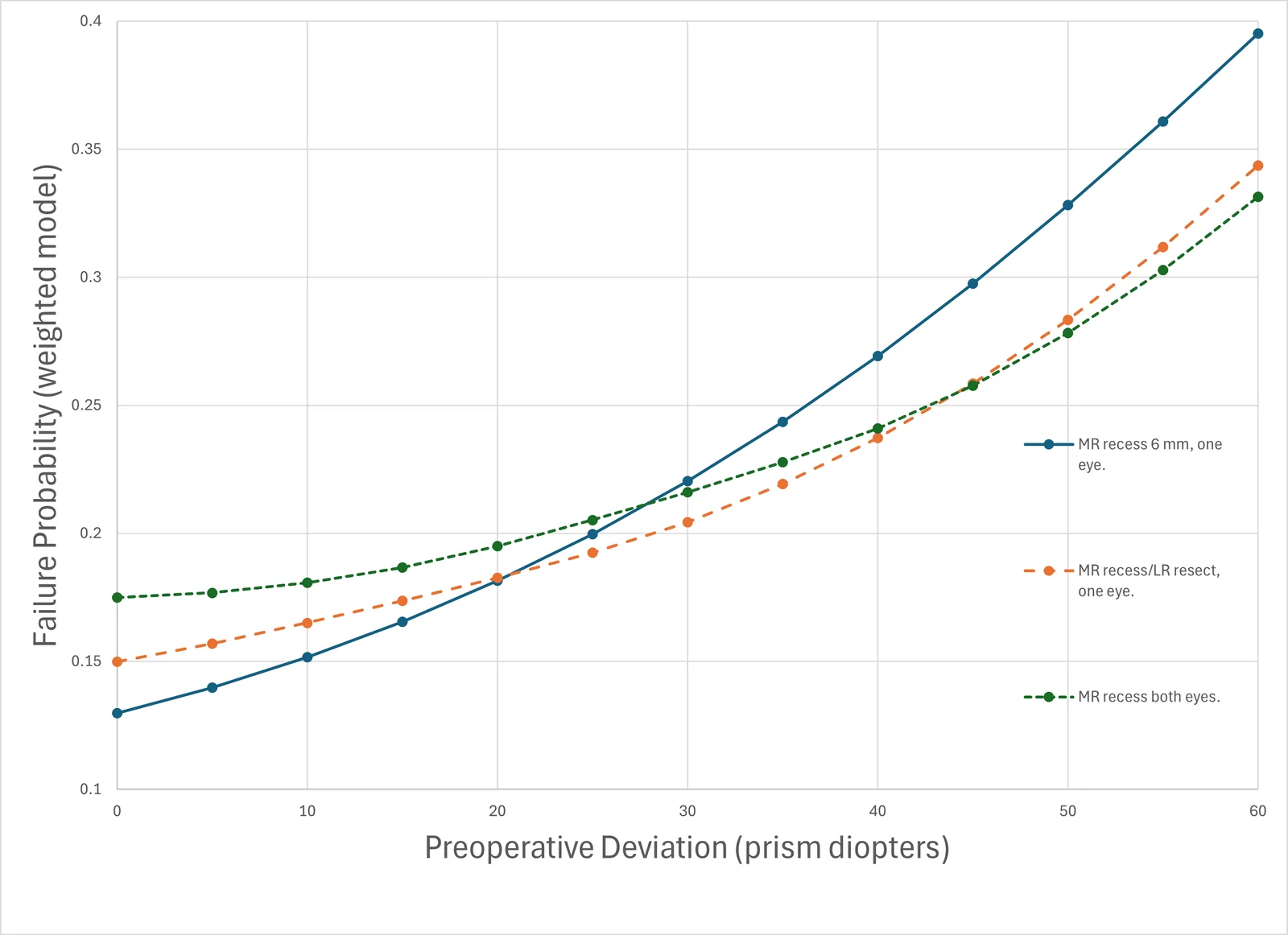
Failure probability as a function of preoperative deviation, for each surgical strategy (unilateral medial rectus recession, unilateral medial rectus recession with lateral rectus resection, and bi-medial recession) This is the model with weighting, so that under-corrections are considered only 64.5% as likely as over-corrections to result in significant adverse effects, in order to reproduce the bi-medial recession strabismus table of Parks (1975). The mm of surgery for each strategy was selected to minimize the weighted failure rate: medial rectus recession one eye of 6 mm and medial rectus recession/lateral rectus and bi-medial recession optimal dosages as in Table 5 and Table 6.

According to the regression equations, small preoperative deviations are optimally treated with a large unilateral medial rectus recession. One caution is that there are limited data with this approach for the smallest deviations. For instance, there were at least 40 cases for which a medial rectus recession of at least 6 mm was performed for a preoperative deviation of 16 prism diopters or less, and for which a recession of 5 mm was performed for a preoperative deviation of 12 prism diopters or less (Table 7). If one performs a large recession for smaller deviations, one is venturing into territory with limited data. 

Thyroid eye disease

In a multivariable model that included 103 cases of thyroid eye disease, the rates of under-correction (p=0.44) and over-correction (p=0.35) were not significantly associated with the surgical dose (Table 8). This lack of association was likely due to the small number of thyroid cases.

**Table 8:** Multivariable logistic predictors of under-corrections and over-corrections after strabismus surgery for esotropia (includes cases of thyroid disease) The variable “thyroid eye disease” equals 1 if thyroid eye disease is present and 0 otherwise. The variable “binocular surgery” equals 1 for binocular surgery and 0 for monocular surgery. n=3369 cases, including 67 with adjustable sutures and 103 with thyroid eye disease Probability of under-correction = 1 / (1 + exp(-(-1.428055+0.056611*(preoperative deviation in degrees) -0.270096*(binocular surgery)-0.064695*(number of muscles operated)-0.367631*(thyroid disease) -0.026136*(mm of surgery if thyroid disease) -0.051293*(mm of surgery if not thyroid disease) ))) Probability of over-correction = 1 / (1 + exp(-(-4.183356-0.031800*(preoperative deviation in degrees) +0.597680*(binocular surgery)+0.463199*(number of muscles operated) -1.002918*(thyroid disease) + 0.061185*(mm surgery if thyroid disease) + 0.079007*(mm surgery if not thyroid disease) )))

Variable	Under-corrections				Over-corrections			
	Odds ratio			p value	Odds ratio			p value
	Estimate	Lower 95%	Upper 95%		Estimate	Lower 95%	Upper 95%	
Preoperative deviation (°)	1.06	1.05	1.07	<0.0001	0.97	0.95	0.99	0.001
Surgical dose (mm) if thyroid disease	0.97	0.91	1.04	0.44	1.06	0.94	1.21	0.35
Surgical dose (mm) if non-thyroid	0.95	0.91	0.99	0.01	1.08	1.01	1.16	0.02
Thyroid eye disease	0.69	0.24	2.01	0.50	0.37	0.03	4.98	0.45
Number of muscles operated	0.94	0.73	1.20	0.61	1.59	1.007	2.36	0.02
Binocular surgery	0.76	0.62	0.93	0.009	1.82	1.23	2.70	0.003

Adjustable sutures

Adjustable sutures were used for 216 cases. For this analysis, the outcome was evaluated post-adjustment. Only six over-corrections (2.8%) and 28 under-corrections (13.0%) were observed. Patients who were under-corrected did not differ according to whether surgery was binocular (p=0.28), by total mm of surgery (p=0.38), the presence of thyroid eye disease (p=0.51), or number of muscles operated (p=0.81). Likewise, the preoperative deviation in under-corrected patients (18.8°, SD: 7.7°) was not significantly different than that of other patients (15.8°, SD: 8.8°, p=0.07).

## Discussion

This study identified predictors of under- and over-correction following horizontal muscle surgery for esotropia and provided insight into the model-predicted optimal surgical strategy.

As one might expect, binocular surgery (as compared with monocular surgery) is less likely to result in under-corrections and more likely to result in over-corrections.

An age-old question in strabismus surgery has been the degree to which a change in the surgical dose (in mm) changes the final alignment. As early as 1951, Scobee noted that “The amount of surgical correction obtained in a patient with esotropia is usually directly proportional to the deviation present before surgery and is not particularly related to the amount of surgery performed as measured in millimeters” [1]. This point has been emphasized more recently [3].

Nonetheless, our study demonstrated a significant dose-response association: larger surgical doses were associated with fewer under-corrections and more over-corrections, even after accounting for preoperative deviation and surgical approach. The difficulty of demonstrating the dose-response curve for strabismus surgery has led to this curve being called a “legend” [3]. Our study sheds light on why the relationship is difficult to demonstrate. In short, strabismus surgery typically “works.” Failures are rare. For instance, in our study, only 7% of surgeries resulted in over-corrections. Therefore, a study of 100 patients with strabismus might only be expected to produce seven over-corrections. In order to learn when failures occur, one needs to analyze thousands of cases.

The regression equations permit determination of model-predicted optimal surgical strategies. For a variety of model assumptions, large medial rectus recessions (about 6 mm in this dataset) were predicted to minimize the failure rate. The idea that a muscle should be operated aggressively, or not at all, is not new. Prangen wrote in 1947 that he performed maximal resections and that “The medial rectus muscle I recess 5 mm, no more and no less” [14]. In 1951, Stine advocated recessing the muscle to the equator and wrote: “If one has decided to attack a muscle, one should either recess or resect it fully” [15]. Also in 1951, Scobee published a series of patients having 6 mm medial rectus recessions, regardless of whether surgery was unilateral or bilateral [1]. According to our results, this “all-or-nothing” approach to each muscle is favored over the tables outlined in Parks in 1975, with graded doses and two-muscle surgery for all cases [2].

The models predicted that as the preoperative deviation increases, one progresses from a unilateral medial rectus recession, to a unilateral two-muscle surgery, and finally to bi-medial recessions. In the models, increasing preoperative deviation generally shifted the model-predicted optimal strategy toward operating on more muscles rather than increasing the per-muscle dose. The precise cutoffs where one transitions to the next surgical approach depend on how one values under-corrections relative to over-corrections. Nonetheless, some common features were seen. For instance, preoperative deviations of 45-50 prism diopters were (according to the models) optimally treated with bi-medial recessions of 6 mm, over a range of model assumptions.

Over-corrections were uncommon in this dataset, and the fact that they were not strongly dependent on surgical dose could indicate that over-corrections occur by a different mechanism, such as by slipped muscles [16], by stretched scars [17], or by variation in the brain’s response to the operation.

The results of this large dataset do not definitively settle the question of optimal surgical approach, because patients and doctors might vary in their preferences. For instance, some surgeons might find over-corrections more objectionable than under-corrections. Moreover, some surgeons might require a certain absolute benefit before being willing to operate on an additional muscle. In addition, when one eye has much better vision than the other, a unilateral surgery might be preferred.

Certain types of restrictive strabismus undoubtedly have a different dose-response than non-restrictive strabismus. It should be emphasized that thyroid disease was not part of the primary analysis and was only included in supplemental analyses. A relatively small number of patients with Duane syndrome were included in the primary analysis, but their responses did not vary significantly from other patients in the multivariable analyses. Moreover, the Duane syndrome subgroup, due to its small size, was unlikely to substantially impact the primary analysis.

This study has multiple limitations. The study population consisted predominantly of children, and additional analyses incorporating larger numbers of adults would be valuable. Nonetheless, the 368 adult procedures included in the primary analysis exceed the adult sample sizes reported in many previously published studies. Larger datasets containing greater numbers of patients with thyroid eye disease or adjustable suture procedures could also help clarify dose-response relationships and define the most effective surgical strategies for these subgroups. We did not present models with separate regression terms for recession and resection amounts because variables distinguishing these effects were not statistically significant in multivariable analyses, although such differences might become apparent in larger cohorts. Follow-up duration was analyzed as a dichotomous variable based on whether it was at least six months; future studies with larger sample sizes may be able to characterize the relationship between follow-up interval and surgical failure in greater detail. Furthermore, outcome classification was based on alignment in primary gaze at distance and near fixation. However, the analysis indirectly incorporated other clinically important outcomes by considering any reoperation a failure. As a result, procedures requiring additional surgery because of diplopia, incomitance, cosmetic concerns, or other postoperative issues were classified as unsuccessful. Finally, data were pooled across multiple surgeons, as evaluation of competing surgical strategies requires a sufficiently large dataset encompassing variability in operative decision-making. For example, determining the preferred management of a 25 prism diopter deviation requires comparison of both one- and two-muscle procedures performed with a range of surgical doses. Although a similar analysis could be conducted using the experience of a single high-volume surgeon who frequently varied surgical technique and dose for similar patients, such a study would face the complementary limitation of uncertain generalizability to other surgeons.

Causal effects would be most rigorously evaluated in large, multicenter controlled trials that randomized patients to varying surgical doses (in mm), numbers of muscles operated, and monocular versus binocular surgery. However, such trials would pose substantial logistical and ethical challenges and are unlikely to be conducted. Consequently, observational data may represent the most practical evidence available for comparing these surgical strategies across the broad range of deviations encountered in clinical practice.

Publication bias could have influenced the observed associations. The analysis necessarily included only reports providing sufficiently detailed individual patient-level data to relate preoperative deviation and surgical characteristics to postoperative outcome. If studies providing such data differed systematically from the broader esotropia surgical literature in patient characteristics, surgical practices, era, or outcomes, selection bias could have resulted, although the direction and magnitude of any such bias are unknown.

Because the analysis pooled published reports spanning 1940-2025, unmeasured differences among studies in patient selection, surgical technique, outcome assessment, follow-up, and practice patterns over time could have influenced the observed associations.

Missing data for age (12% of cases) or follow-up reduced the sample sizes for analyses incorporating these variables and could introduce bias if the availability of these data was associated with surgical outcomes.

Because individual patients were pooled from published case series, outcomes within a given publication may have been correlated because of shared surgeons, surgical techniques, patient populations, follow-up protocols, and outcome assessment; the regression models did not account for clustering by publication, which could affect the estimated precision of the associations.

The dataset included heterogeneous forms of esotropia in both children and adults. Specific etiologies were not consistently reported, particularly in older studies, and most diagnostic subgroups were not sufficiently large or consistently defined to support reliable separate multivariable models. We therefore did not model most esotropia subtypes separately.

## Conclusions

This large pooled analysis of individual patient retrospective data demonstrates that surgical dose has an independent relationship with outcome after esotropia surgery: increasing the surgical dose was associated with a decrease in the risk of under-correction and an increased risk of over-correction, even after accounting for preoperative deviation and surgical approach. Thus, larger surgical doses were associated with a larger response, although the magnitude of the dose-response relationship is relatively modest. Models designed to minimize surgical failure generally favored large recessions or resections of the muscles selected for surgery, with progressively more muscles operated as the preoperative deviation increased. This approach more closely resembles the strategy advocated by Scobee than the traditional strategy of using two-muscle surgery with progressively graded doses described by Parks. However, the model-predicted optimal surgical strategy depends on the relative importance assigned to under- and over-correction, the additional risks of operating on more muscles, and individual patient characteristics. Modeled strategies depend on assumptions about outcome weighting and data availability. These findings support considering both the per-muscle surgical dose and the number of muscles operated when planning esotropia surgery, rather than basing treatment on a graded dose-response table alone.
